# Supplementary material for: Complexome profiling of the Chlamydomonas psb28 mutant reveals TEF5 as an early PSII assembly factor
Source: Plant Cell. 2025 Mar 17;37(6):koaf055. doi: 10.1093/plcell/koaf055 (PMC12164590; doi:10.1093/plcell/koaf055)
Supplement: koaf055_Supplementary_Data [file koaf055_supplementary_data.zip › psb28_interactive.zip!/plots/intensity/qualityPlots/peptides/_Cre12.g537550.t1.1.html]

### Cre12.g537550.t1.1

Trivial name:   
SeqCoverage [median]: 14.20  
Mass: 36.81 kDa  
  
 

| Sequence | MissedCleavage | Mass [Da] | wtIntensity | muIntensity | Score |
| --- | --- | --- | --- | --- | --- |
| AAQLSPTYAFAVANR | 0 | 1578.82 | 0.00 | 5198.24 | 126.19 |
| AVALFEVGR | 0 | 960.54 | 0.00 | 2727.01 | 74.13 |
| AVALFEVGRTEEAIR | 1 | 1659.89 | 0.00 | 707.11 | 34.18 |
| FEEAVSDYR | 0 | 1114.49 | 0.00 | 954.35 | 101.64 |
| RYPDFADMR | 1 | 1169.53 | 0.00 | 477.17 | 67.11 |
|  |  |  | 0.0 | 10063.9 |  |
